# Supplementary material for: Estimating Vaccine Confidence Levels among Healthcare Staff and Students of a Tertiary Institution in South Africa
Source: Vaccines (Basel). 2021 Oct 27;9(11):1246. doi: 10.3390/vaccines9111246 (PMC8618030; doi:10.3390/vaccines9111246)
Supplement: Supplementary file 1 [file vaccines-09-01246-s001.zip › Table S1 Associations between categorical demographic variables and the importance of vaccines for children statement.pdf]

Table S1. Associations between categorical demographic variables and the importance of vaccines for children statement.

Categorical demographic variables		Vaccines are important for children to have statement						p-value
		Disagree		Agree		Total		
		Count	Row N %	Count	Row N %	Count	Row N %	
Staff/Student	Staff	4	1.6%	245	98.4%	249	100.0%	0.472
	Student	19	2.9%	644	97.1%	663	100.0%	
	Both	3	3.6%	80	96.4%	83	100.0%	
	Total	26	2.6%	969	97.4%	995	100.0%	
Sex	Male	9	3.6%	241	96.4%	250	100.0%	0.376
	Female	17	2.3%	727	97.7%	744	100.0%	
	Other	0	0.0%	1	100.0%	1	100.0%	
	Total	26	2.6%	969	97.4%	995	100.0%	
degree	BSc	10	2.8%	345	97.2%	355	100.0%	0.786
	Hons	5	3.8%	128	96.2%	133	100.0%	
	MBBS	6	2.8%	212	97.2%	218	100.0%	
	MSc	3	1.5%	196	98.5%	199	100.0%	
	PhD	2	2.2%	88	97.8%	90	100.0%	
	Total	26	2.6%	969	97.4%	995	100.0%	
religion	Islam	7	6.4%	103	93.6%	110	100.0%	0.351
	Roman Catholic	3	3.3%	88	96.7%	91	100.0%	
	Orthodox	10	3.2%	303	96.8%	313	100.0%	
	Pentecostal	3	1.6%	186	98.4%	189	100.0%	
	Traditional	2	2.7%	73	97.3%	75	100.0%	
	Jewish	0	0.0%	7	100.0%	7	100.0%	
	Buddhist	0	0.0%	4	100.0%	4	100.0%	
	Hindu	0	0.0%	25	100.0%	25	100.0%	
	Atheist	0	0.0%	74	100.0%	74	100.0%	
	Agnostic	1	1.4%	72	98.6%	73	100.0%	
	Other	0	0.0%	25	100.0%	25	100.0%	
	7th Day Adventist	0	0.0%	9	100.0%	9	100.0%	
	Total	26	2.6%	969	97.4%	995	100.0%	
Age group	≤24	13	3.2%	394	96.8%	407	100.0%	0.457
	25-34	3	1.2%	238	98.8%	241	100.0%	
	35-44	5	2.7%	183	97.3%	188	100.0%	
	45-54	2	2.5%	78	97.5%	80	100.0%	

	55-64	2	2.9%	67	97.1%	69	100.0%	
	≥65	1	10.0%	9	90.0%	10	100.0%	
	Total	26	2.6%	969	97.4%	995	100.0%	
