# Supplementary material for: Estimating Vaccine Confidence Levels among Healthcare Staff and Students of a Tertiary Institution in South Africa
Source: Vaccines (Basel). 2021 Oct 27;9(11):1246. doi: 10.3390/vaccines9111246 (PMC8618030; doi:10.3390/vaccines9111246)
Supplement: Supplementary file 1 [file vaccines-09-01246-s001.zip › Table S10 The association between quantitative variables and belief in religious compatibility of vaccines.pdf]

Table S10: The association between quantitative variables and belief in religious compatibility of vaccines

Quantitative variables		Vaccines are compatible with my religious beliefs			p-value
		Disagree	Agree	Total	
Age	Median	33,00	29,00	29,00	0.267
	Percentile 25	22,00	22,00	22,00	
	Percentile 75	43,50	38,00	38,00	
Post matric	Median	5,50	6,00	6,00	0.614
	Percentile 25	3,00	4,00	4,00	
	Percentile 75	12,50	11,00	11,00	
