# Supplementary material for: Estimating Vaccine Confidence Levels among Healthcare Staff and Students of a Tertiary Institution in South Africa
Source: Vaccines (Basel). 2021 Oct 27;9(11):1246. doi: 10.3390/vaccines9111246 (PMC8618030; doi:10.3390/vaccines9111246)
Supplement: Supplementary file 1 [file vaccines-09-01246-s001.zip › Table S11 Associations between categorical demographic variables and intention to receive COVID 19 vaccine.pdf]

Table S11: Associations between categorical demographic variables and intention to receive COVID 19 vaccine

Categorical demographic variables		I will take a Covid-19 vaccine when one becomes available						p-value
		Disagree		Agree		Total		
		Count	Row N %	Count	Row N %	Count	Row N %	
Staff/Student	Staff	19	8.3%	210	91.7%	229	100.0%	0.383
	Student	63	11.1%	504	88.9%	567	100.0%	
	Both	10	13.0%	67	87.0%	77	100.0%	
	Total	92	10.5%	781	89.5%	873	100.0%	
Sex	Male	20	8.5%	216	91.5%	236	100.0%	0.317
	Female	70	11.0%	565	89.0%	635	100.0%	
	Other	2	100.0%	0	0.0%	2	100.0%	
	Total	92	10.5%	781	89.5%	873	100.0%	
degree	BSc	35	11.7%	263	88.3%	298	100.0%	0.161
	Hons	18	16.1%	94	83.9%	112	100.0%	
	MBBS	18	9.0%	182	91.0%	200	100.0%	
	MSc	15	8.5%	162	91.5%	177	100.0%	
	PhD	6	7.0%	80	93.0%	86	100.0%	
	Total	92	10.5%	781	89.5%	873	100.0%	
religion	Islam	7	7.5%	86	92.5%	93	100.0%	.
	Roman Catholic	11	12.8%	75	87.2%	86	100.0%	
	Orthodox	31	11.6%	237	88.4%	268	100.0%	
	Pentecostal	22	13.7%	139	86.3%	161	100.0%	
	Traditional	7	11.1%	56	88.9%	63	100.0%	
	Jewish	1	14.3%	6	85.7%	7	100.0%	
	Buddhist	1	25.0%	3	75.0%	4	100.0%	
	Hindu	0	0.0%	24	100.0%	24	100.0%	
	Atheist	4	5.6%	68	94.4%	72	100.0%	
	Agnostic	2	2.9%	66	97.1%	68	100.0%	
	Other	2	10.0%	18	90.0%	20	100.0%	
	7th Day Adventist	4	57.1%	3	42.9%	7	100.0%	
	Total	92	10.5%	781	89.5%	873	100.0%	
Age group	≤24	38	10.9%	311	89.1%	349	100.0%	0.992
	25-34	20	9.8%	185	90.2%	205	100.0%	
	35-44	18	10.8%	148	89.2%	166	100.0%	
	45-54	7	9.3%	68	90.7%	75	100.0%	

	55-64	8	11.9%	59	88.1%	67	100.0%	
	≥65	1	9.1%	10	90.9%	11	100.0%	
	Total	92	10.5%	781	89.5%	873	100.0%	
