# Supplementary material for: Estimating Vaccine Confidence Levels among Healthcare Staff and Students of a Tertiary Institution in South Africa
Source: Vaccines (Basel). 2021 Oct 27;9(11):1246. doi: 10.3390/vaccines9111246 (PMC8618030; doi:10.3390/vaccines9111246)
Supplement: Supplementary file 1 [file vaccines-09-01246-s001.zip › Table S12 Associations between quantitative variables and intention to receive COVID 19 vaccine.pdf]

Table S12: Associations between quantitative variables and intention to receive COVID 19 vaccine

Quantitative variables		I will take a Covid-19 vaccine when one becomes available			p-value
		Disagree	Agree	Total	
Age	Median	30,00	30,00	30,00	0.990
	Percentile 25	21,00	22,00	22,00	
	Percentile 75	38,00	39,00	38,00	
Post matric	Median	5,00	6,00	6,00	0.301
	Percentile 25	3,00	4,00	4,00	
	Percentile 75	12,00	11,00	11,00	
