# Supplementary material for: Estimating Vaccine Confidence Levels among Healthcare Staff and Students of a Tertiary Institution in South Africa
Source: Vaccines (Basel). 2021 Oct 27;9(11):1246. doi: 10.3390/vaccines9111246 (PMC8618030; doi:10.3390/vaccines9111246)
Supplement: Supplementary file 1 [file vaccines-09-01246-s001.zip › Table S13a Association between levels of education, vaccine confidence statements and the intention to receive a COVID 19 vaccine.pdf]

**Table S13a:** Association between levels of education, vaccine confidence statements and the intention to receive a COVID 19 vaccine

| Statements | Undergraduates (BSc. & MBBS) | | Postgraduates (Honours & above) | | Pearson's chi square 2-sided p-value |
|---|---|---|---|---|---|
| | Agree | Disagree | Agree | Disagree | |
| 1. Vaccines are important for children to have | 92.2% | 2.8% | 97.6% | 2.4% | 0.841 |
| 2. Vaccines are important for me to have | 95.1% | 4.9% | 95.9% | 4.1% | 0.664 |
| 3. Overall, I think vaccines are safe | 95.9% | 4.1% | 94.7% | 5.3% | 0.431 |
| 4. Overall, I think vaccines are effective | 98.0% | 2.0% | 96.6% | 3.4% | 0.218 |
| 5. Vaccines are compatible with my religious beliefs | 96.1% | 3.9% | 96.1% | 96.1% | 1.000 |
| 6. I will take a Covid-19 vaccine when one becomes available | 89.4% | 10.6% | 89.6% | 10.4% | 1.000 |
