# Supplementary material for: Estimating Vaccine Confidence Levels among Healthcare Staff and Students of a Tertiary Institution in South Africa
Source: Vaccines (Basel). 2021 Oct 27;9(11):1246. doi: 10.3390/vaccines9111246 (PMC8618030; doi:10.3390/vaccines9111246)
Supplement: Supplementary file 1 [file vaccines-09-01246-s001.zip › Table S13b Association between levels of education, vaccine confidence statements and the intention to receive a COVID 19.pdf]

Table S 13b: Association between levels of education, vaccine confidence statements and the intention to receive a COVID 19

Statements	Up to Honors (BSc., MBBS, & Honors)		Masters & above (Masters & Ph.D.)		p-value
	Agree	Disagree	Agree	Disagree	
1. Vaccines are important for children to have	97.0%	3.0%	98.3%	1.7%	0.381
2. Vaccines are important for me to have	94.5%	5.5%	97.6%	2.4%	0.043
3. Overall, I think vaccines are safe	95.2%	4.8%	96.0%	4.6%	0.612
4. Overall, I think vaccines are effective	97.2%	2.8%	97.9%	2.1%	0.660
5. Vaccines are compatible with my religious beliefs	95.6%	4.4%	97.3%	2.7%	0.262
6. I will take a Covid-19 vaccine when one becomes available	88.4%	11.6%	92.0%	8.0%	0.119
