# Supplementary material for: Estimating Vaccine Confidence Levels among Healthcare Staff and Students of a Tertiary Institution in South Africa
Source: Vaccines (Basel). 2021 Oct 27;9(11):1246. doi: 10.3390/vaccines9111246 (PMC8618030; doi:10.3390/vaccines9111246)
Supplement: Supplementary file 1 [file vaccines-09-01246-s001.zip › Table S2 Associations between quantitative variables and importance of vaccines for children statement.pdf]

Table S2: Associations between quantitative variables and importance of vaccines for children statement

Quantitative variables		Vaccines are important for children to have statement			
		Disagree	Agree	Total	p-value
age	Median	24.00	29.00	29.00	0.523
	Percentile 25	20.00	22.00	22.00	
	Percentile 75	39.00	38.00	38.00	
Post matric years of schooling	Median	5.50	6.00	6.00	0.572
	Percentile 25	3.00	4.00	4.00	
	Percentile 75	13.00	11.00	11.00	
