# Supplementary material for: Estimating Vaccine Confidence Levels among Healthcare Staff and Students of a Tertiary Institution in South Africa
Source: Vaccines (Basel). 2021 Oct 27;9(11):1246. doi: 10.3390/vaccines9111246 (PMC8618030; doi:10.3390/vaccines9111246)
Supplement: Supplementary file 1 [file vaccines-09-01246-s001.zip › Table S3 Associations between categorical demographic variables and importance of vaccines for self statement.pdf]

Table S3: Associations between categorical demographic variables and importance of vaccines for self statement

Categorical demographic variables		Vaccines are important for me to have statement						p-value
		Disagree		Agree		Total		
		Count	Row N %	Count	Row N %	Count	Row N %	
Staff/Student	Staff	10	4.0%	238	96.0%	248	100.0%	0.882
	Student	31	4.7%	626	95.3%	657	100.0%	
	Both	4	5.0%	76	95.0%	80	100.0%	
	Total	45	4.6%	940	95.4%	985	100.0%	
Sex	Male	9	3.6%	240	96.4%	249	100.0%	0.485
	Female	36	4.9%	700	95.1%	736	100.0%	
	Total	45	4.6%	940	95.4%	985	100.0%	
degree	BSc	19	5.4%	334	94.6%	353	100.0%	0.140
	Hons	10	7.8%	119	92.2%	129	100.0%	
	MBBS	9	4.2%	206	95.8%	215	100.0%	
	MSc	4	2.0%	194	98.0%	198	100.0%	
	PhD	3	3.3%	87	96.7%	90	100.0%	
	Total	45	4.6%	940	95.4%	985	100.0%	
religion	Islam	7	6.5%	101	93.5%	108	100.0%	
	Roman Catholic	4	4.3%	88	95.7%	92	100.0%	
	Orthodox	12	3.9%	295	96.1%	307	100.0%	
	Pentecostal	10	5.3%	179	94.7%	189	100.0%	
	Traditional	4	5.6%	68	94.4%	72	100.0%	
	Jewish	0	0.0%	7	100.0%	7	100.0%	
	Buddhist	0	0.0%	4	100.0%	4	100.0%	
	Hindu	0	0.0%	25	100.0%	25	100.0%	
	Atheist	2	2.6%	74	97.4%	76	100.0%	
	Agnostic	2	2.8%	70	97.2%	72	100.0%	
	Other	1	4.2%	23	95.8%	24	100.0%	
	7th Day Adventist	3	33.3%	6	66.7%	9	100.0%	
	Total	45	4.6%	940	95.4%	985	100.0%	
Age group	≤24	18	4.5%	386	95.5%	404	100.0%	0.463
	25-34	8	3.4%	225	96.6%	233	100.0%	
	35-44	10	5.2%	181	94.8%	191	100.0%	
	45-54	3	3.8%	77	96.3%	80	100.0%	
	55-64	6	9.0%	61	91.0%	67	100.0%	

	≥65	0	0.0%	10	100.0%	10	100.0%	
	Total	45	4.6%	940	95.4%	985	100.0%	
