# Supplementary material for: Estimating Vaccine Confidence Levels among Healthcare Staff and Students of a Tertiary Institution in South Africa
Source: Vaccines (Basel). 2021 Oct 27;9(11):1246. doi: 10.3390/vaccines9111246 (PMC8618030; doi:10.3390/vaccines9111246)
Supplement: Supplementary file 1 [file vaccines-09-01246-s001.zip › Table S4 Associations between quantitative variables and importance of vaccines for self statement.pdf]

Table S4: Associations between quantitative variables and importance of vaccines for self statement

Quantitative variables		Vaccines are important for me to have statement			p-value
		Disagree	Agree	Total	
age	Median	32,00	29,00	29,00	0.429
	Percentile 25	22,00	21,00	22,00	
	Percentile 75	39,00	38,00	38,00	
Post matric	Median	5,00	6,00	6,00	0.611
	Percentile 25	3,00	4,00	4,00	
	Percentile 75	14,00	11,00	11,00	
