# Supplementary material for: Estimating Vaccine Confidence Levels among Healthcare Staff and Students of a Tertiary Institution in South Africa
Source: Vaccines (Basel). 2021 Oct 27;9(11):1246. doi: 10.3390/vaccines9111246 (PMC8618030; doi:10.3390/vaccines9111246)
Supplement: Supplementary file 1 [file vaccines-09-01246-s001.zip › Table S5 Associations between categorical demographic variables and vaccine safety statement.pdf]

Table S5: Associations between categorical demographic variables and vaccine safety statement

Categorical demographic variables		Overall, I think vaccines are safe						p-value
		Disagree		Agree		Total		
		Count	Row N %	Count	Row N %	Count	Row N %	
Staff/Student	Staff	7	3.0%	227	97.0%	234	100.0%	0.379
	Student	31	5.0%	592	95.0%	623	100.0%	
	Both	5	6.2%	76	93.8%	81	100.0%	
	Total	43	4.6%	895	95.4%	938	100.0%	
Sex	Male	6	2.5%	234	97.5%	240	100.0%	0.118
	Female	37	5.3%	660	94.7%	697	100.0%	
	Other	0	0.0%	1	100.0%	1	100.0%	
	Total	43	4.6%	895	95.4%	938	100.0%	
degree	BSc	15	4.6%	311	95.4%	326	100.0%	0.285
	Hons	10	8.3%	110	91.7%	120	100.0%	
	MBBS	7	3.3%	207	96.7%	214	100.0%	
	MSc	7	3.7%	181	96.3%	188	100.0%	
	PhD	4	4.4%	86	95.6%	90	100.0%	
	Total	43	4.6%	895	95.4%	938	100.0%	
religion	Islam	7	6.8%	96	93.2%	103	100.0%	
	Roman Catholic	5	5.7%	82	94.3%	87	100.0%	
	Orthodox	12	4.2%	277	95.8%	289	100.0%	
	Pentecostal	9	5.1%	169	94.9%	178	100.0%	
	Traditional	3	4.3%	66	95.7%	69	100.0%	
	Jewish	0	0.0%	8	100.0%	8	100.0%	
	Buddhist	0	0.0%	4	100.0%	4	100.0%	
	Hindu	0	0.0%	25	100.0%	25	100.0%	
	Atheist	2	2.7%	72	97.3%	74	100.0%	
	Agnostic	1	1.4%	69	98.6%	70	100.0%	
	Other	1	4.5%	21	95.5%	22	100.0%	
	7th Day Adventist	3	33.3%	6	66.7%	9	100.0%	
	Total	43	4.6%	895	95.4%	938	100.0%	
Age group	≤24	19	5.0%	364	95.0%	383	100.0%	0.746
	25-34	8	3.6%	217	96.4%	225	100.0%	
	35-44	8	4.4%	172	95.6%	180	100.0%	
	45-54	3	4.0%	72	96.0%	75	100.0%	
	55-64	5	7.7%	60	92.3%	65	100.0%	

	≥65	0	0.0%	10	100.0%	10	100.0%	
	Total	43	4.6%	895	95.4%	938	100.0%	
