# Supplementary material for: Estimating Vaccine Confidence Levels among Healthcare Staff and Students of a Tertiary Institution in South Africa
Source: Vaccines (Basel). 2021 Oct 27;9(11):1246. doi: 10.3390/vaccines9111246 (PMC8618030; doi:10.3390/vaccines9111246)
Supplement: Supplementary file 1 [file vaccines-09-01246-s001.zip › Table S6 Associations between quantitative variables and vaccine safety statement.pdf]

Table S6: Associations between quantitative variables and vaccine safety statement

Quantitative variables		Overall, I think vaccines are safe			p-value
		Disagree	Agree	Total	
Age	Median	27,00	29,00	29,00	0.994
	Percentile 25	21,00	22,00	22,00	
	Percentile 75	42,00	38,00	38,00	
Post matric	Median	5,00	6,00	6,00	0.323
	Percentile 25	3,00	4,00	4,00	
	Percentile 75	13,00	11,00	11,00	
