# Supplementary material for: Estimating Vaccine Confidence Levels among Healthcare Staff and Students of a Tertiary Institution in South Africa
Source: Vaccines (Basel). 2021 Oct 27;9(11):1246. doi: 10.3390/vaccines9111246 (PMC8618030; doi:10.3390/vaccines9111246)
Supplement: Supplementary file 1 [file vaccines-09-01246-s001.zip › Table S7 Associations between categorical demographic variables and vaccine effectiveness statement.pdf]

Table S7: Associations between categorical demographic variables and vaccine effectiveness statement

Categorical demographic variables		Overall, I think vaccines are effective						p-value
		Disagree		Agree		Total		
		Count	Row N %	Count	Row N %	Count	Row N %	
Staff/Student	Staff	5	2.1%	235	97.9%	240	100.0%	0.109
	Student	15	2.3%	629	97.7%	644	100.0%	
	Both	5	6.1%	77	93.9%	82	100.0%	
	Total	25	2.6%	941	97.4%	966	100.0%	
Sex	Male	3	1.2%	243	98.8%	246	100.0%	0.183
	Female	22	3.1%	697	96.9%	719	100.0%	
	Other	0	0.0%	1	100.0%	1	100.0%	
	Total	25	2.6%	941	97.4%	966	100.0%	
degree	BSc	6	1.8%	333	98.2%	339	100.0%	0.217
	Hons	8	6.3%	119	93.7%	127	100.0%	
	MBBS	5	2.3%	212	97.7%	217	100.0%	
	MSc	4	2.1%	189	97.9%	193	100.0%	
	PhD	2	2.2%	88	97.8%	90	100.0%	
	Total	25	2.6%	941	97.4%	966	100.0%	
religion	Islam	5	4.6%	104	95.4%	109	100.0%	0.109
	Roman Catholic	2	2.2%	89	97.8%	91	100.0%	
	Orthodox	7	2.3%	293	97.7%	300	100.0%	
	Pentecostal	5	2.7%	180	97.3%	185	100.0%	
	Traditional	2	2.9%	66	97.1%	68	100.0%	
	Jewish	0	0.0%	8	100.0%	8	100.0%	
	Buddhist	0	0.0%	4	100.0%	4	100.0%	
	Hindu	0	0.0%	25	100.0%	25	100.0%	
	Atheist	1	1.4%	73	98.6%	74	100.0%	
	Agnostic	1	1.4%	70	98.6%	71	100.0%	
	Other	0	0.0%	22	100.0%	22	100.0%	
	7th Day Adventist	2	22.2%	7	77.8%	9	100.0%	
	Total	25	2.6%	941	97.4%	966	100.0%	
Age group	≤24	8	2.0%	390	98.0%	398	100.0%	0.486
	25-34	7	3.1%	221	96.9%	228	100.0%	
	35-44	4	2.2%	180	97.8%	184	100.0%	
	45-54	2	2.5%	78	97.5%	80	100.0%	
	55-64	4	6.1%	62	93.9%	66	100.0%	

	≥65	0	0.0%	10	100.0%	10	100.0%	
	Total	25	2.6%	941	97.4%	966	100.0%	
