# Supplementary material for: Estimating Vaccine Confidence Levels among Healthcare Staff and Students of a Tertiary Institution in South Africa
Source: Vaccines (Basel). 2021 Oct 27;9(11):1246. doi: 10.3390/vaccines9111246 (PMC8618030; doi:10.3390/vaccines9111246)
Supplement: Supplementary file 1 [file vaccines-09-01246-s001.zip › Table S8 Associations between quantitative variables and vaccine effectiveness statement.pdf]

Table S8: Associations between quantitative variables and vaccine effectiveness statement

Quantitative variables		Overall, I think vaccines are effective			p-value
		Disagree	Agree	Total	
Age	Median	33,00	29,00	29,00	0.236
	Percentile 25	23,00	21,00	22,00	
	Percentile 75	44,00	38,00	38,00	
Post matric	Median	9,00	6,00	6,00	0.200
	Percentile 25	5,00	4,00	4,00	
	Percentile 75	14,00	11,00	11,00	
