# Supplementary material for: Estimating Vaccine Confidence Levels among Healthcare Staff and Students of a Tertiary Institution in South Africa
Source: Vaccines (Basel). 2021 Oct 27;9(11):1246. doi: 10.3390/vaccines9111246 (PMC8618030; doi:10.3390/vaccines9111246)
Supplement: Supplementary file 1 [file vaccines-09-01246-s001.zip › Table S9 Associations between categorical demographic variables and vaccine confidence and belief in religious compatibility of vaccines.pdf]

Table S9: Associations between categorical demographic variables and vaccine confidence and belief in religious compatibility of vaccines

Categorical demographic variables		Vaccines are compatible with my religious beliefs						p-value
		Disagree		Agree		Total		
		Count	Row N %	Count	Row N %	Count	Row N %	
Staff/Student	Staff	12	5.2%	217	94.8%	229	100.0%	0.352
	Student	20	3.3%	592	96.7%	612	100.0%	
	Both	4	5.1%	75	94.9%	79	100.0%	
	Total	36	3.9%	884	96.1%	920	100.0%	
Sex	Male	12	5.2%	220	94.8%	232	100.0%	0.234
	Female	23	3.4%	663	96.6%	686	100.0%	
	Total	35	3.8%	883	96.2%	918	100%	
degree	BSc	16	4.8%	314	95.2%	330	100.0%	0.123
	Hons	8	6.3%	118	93.7%	126	100.0%	
	MBBS	5	2.5%	197	97.5%	202	100.0%	
	MSc	7	3.9%	174	96.1%	181	100.0%	
	PhD	0	0.0%	81	100.0%	81	100.0%	
	Total	36	3.9%	884	96.1%	920	100.0%	
Religion	Islam	3	2.9%	100	97.1%	103	100.0%	0.851
	Roman Catholic	4	5.0%	76	95.0%	80	100.0%	
	Orthodox	15	5.2%	276	94.8%	291	100.0%	
	Pentecostal	7	4.0%	169	96.0%	176	100.0%	
	Traditional	1	1.5%	66	98.5%	67	100.0%	
	Jewish	0	0.0%	7	100.0%	7	100.0%	
	Buddhist	0	0.0%	3	100.0%	3	100.0%	
	Hindu	0	0.0%	24	100.0%	24	100.0%	
	Atheist	4	5.3%	71	94.7%	75	100.0%	
	Agnostic	2	3.0%	64	97.0%	66	100.0%	
	Other	0	0.0%	21	100.0%	21	100.0%	

	7th Day Adventist	0	0.0%	7	100.0%	7	100.0%	
	Total	36	3.9%	884	96.1%	920	100.0%	
Age group	≤24	13	3.5%	363	96.5%	376	100.0%	0.756
	25-34	7	3.2%	213	96.8%	220	100.0%	
	35-44	7	4.0%	166	96.0%	173	100.0%	
	45-54	4	5.3%	71	94.7%	75	100.0%	
	55-64	4	6.2%	61	93.8%	65	100.0%	
	≥65	1	9.1%	10	90.9%	11	100.0%	
	Total	36	3.9%	884	96.1%	920	100.0%	
